# Supplementary material for: Identification and characterization of specific motifs in effector proteins of plant parasites using MOnSTER
Source: Commun Biol. 2024 Jul 12;7:850. doi: 10.1038/s42003-024-06515-9 (PMC11239862; doi:10.1038/s42003-024-06515-9)
Supplement: Supplementary file 2 — supplementary material [file 42003_2024_6515_MOESM2_ESM.pdf]

Supplementary Figures

Supplementary Figure 1: Occurrences and co-occurrences of CLUMPs in positive and negative dataset – Oomycetes.

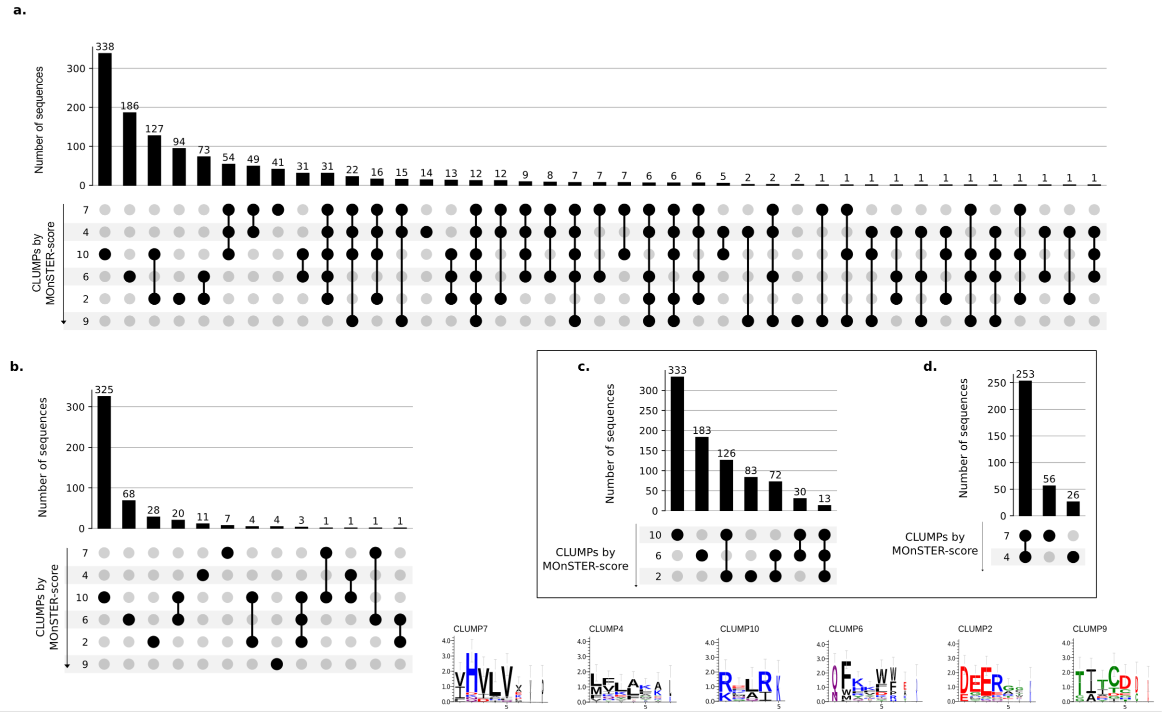

Each upset plot represents the number of sequences in the respective dataset according to the presence of one or more CLUMPs ordered by MOnSTER-score. **(a)** positive dataset, **(b)** negative dataset, (c-d) specifically show the occurrence and co-occurrence of CLUMPs corresponding to RxLR and dEER (CLUMP10, 6, 2), and LxLFLAK-HVLVxxP (CLUMP7,4), in RxLR and Crinkler-effector sequences, respectively. In the bottom right corner, the motif logos of the respective CLUMPs (colors corresponds to 'chemistry' color-scheme in WebLogo3 [1] used to obtain the images).

Supplementary Figure 2: Sequence position preference of motifs in CLUMPs – Oomycetes.

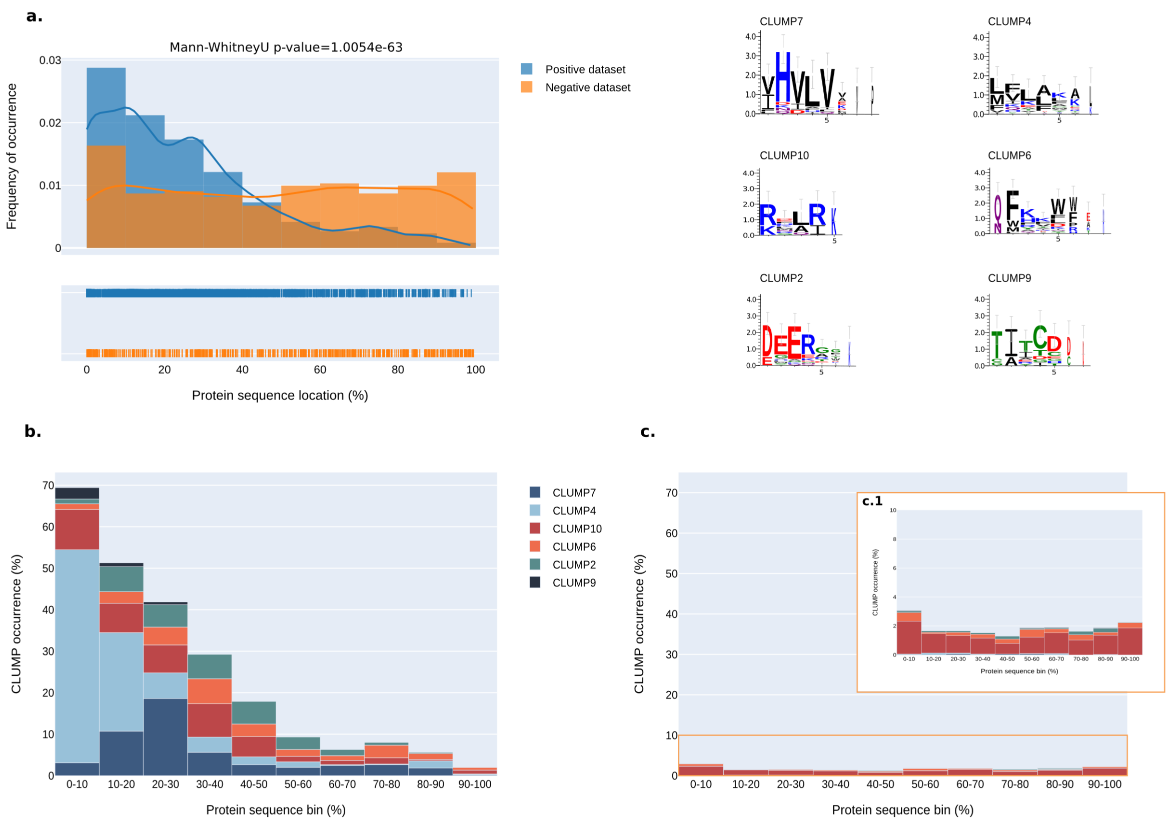

(a) Shows the overall preference position of CLUMPs in the positive (blu trace) compared to the negative (orange trace) dataset. In the rug-plot each line corresponds to a CLUMP-motif position occurrence. **(b)** represents the same distribution in the positive dataset but divided by each CLUMP abundance in the sequence bin. **(c)** shows the same concept but in the negative dataset; **(c.1)** is a zoom-in of the original plot to better visualize the occurrences of CLUMPs. The distributions are all represented in bins of 10% each as absolute positions on the sequence. In the upper right corner, the motif logos of the respective CLUMPs (colors corresponds to 'chemistry' color-scheme in WebLogo3 [1] used to obtain the images).

Supplementary Figure 3: Sequence position preference of motifs in CLUMPs – PPNs.

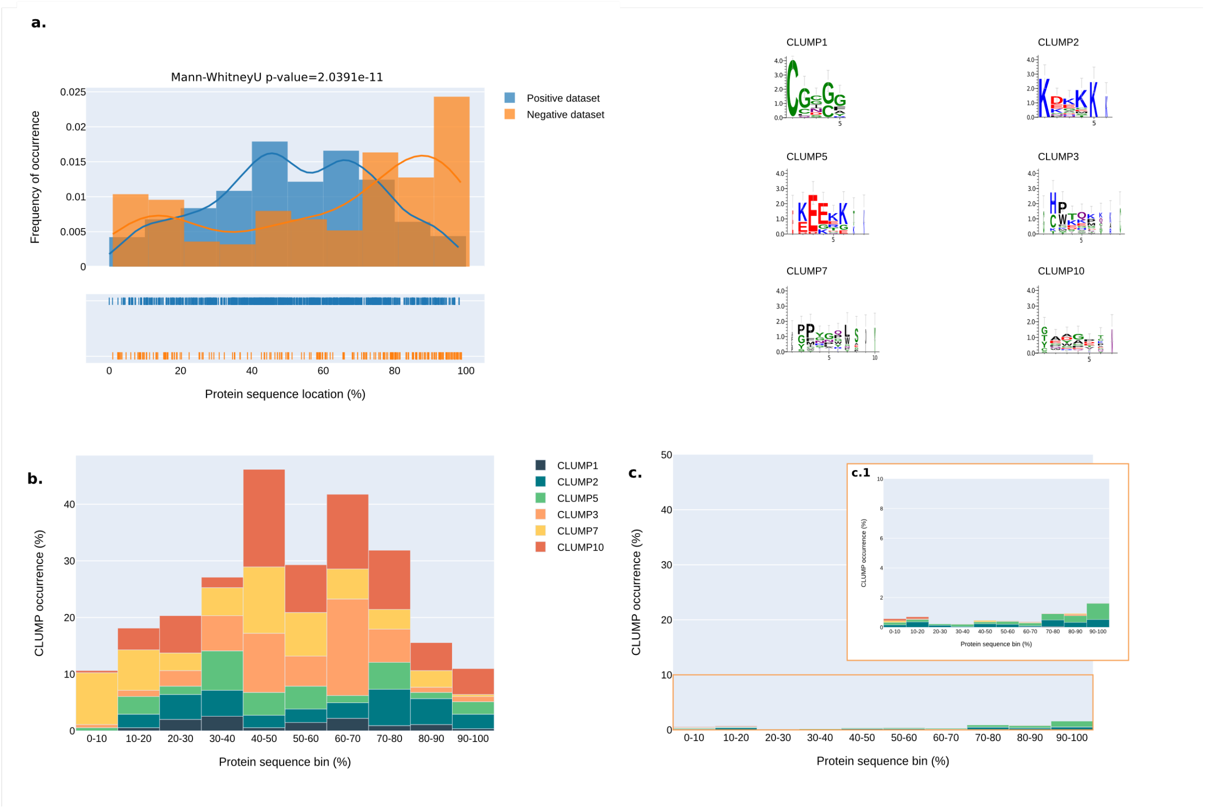

(a) Shows the overall preference of CLUMPs in the positive (blu trace) compared to the negative (orange trace) dataset. In the rug-plot each line corresponds to a CLUMP-motif position occurrence. **(b)** represents the same distribution in the positive dataset but divided by each CLUMP abundance in the sequence bin. **(c)** shows the same concept but in the negative dataset; **(c.1)** is a zoom-in of the original plot to better visualize the occurrences of CLUMPs. The distributions are all represented in bins of 10% each as absolute positions on the sequence. In the upper right corner, the motif logos of the respective CLUMPs (colors corresponds to 'chemistry' color-scheme in WebLogo3 [1] used to obtain the images).

Supplementary Figure 4: Occurrence and co-occurrence of CLUMPs in PPNs.

a.

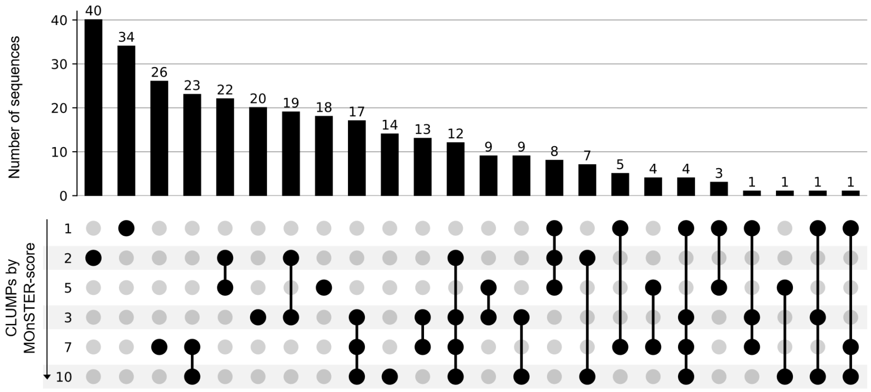

b.

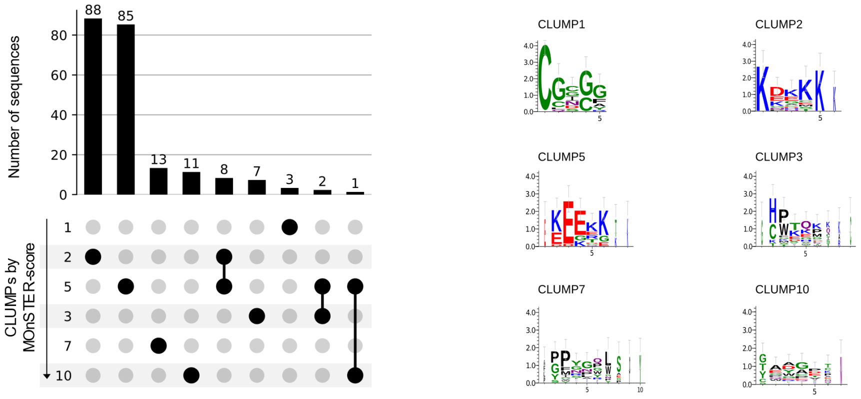

Representation of occurrence and co-occurrence of each best-scoring CLUMP in protein sequences, according to their MOnSTER-score. **(a)** shows the number of sequences and the occurrence of CLUMPs in the positive dataset, while **(b)** represents the same measures in the negative dataset. In the bottom right corner, the motif logos of the respective CLUMPs (colors corresponds to 'chemistry' color-scheme in WebLogo3 [1] used to obtain the images).

Supplementary Figure 5. Occurrence and co-occurrence of CLUMPs in proteins with or without the signal peptide.

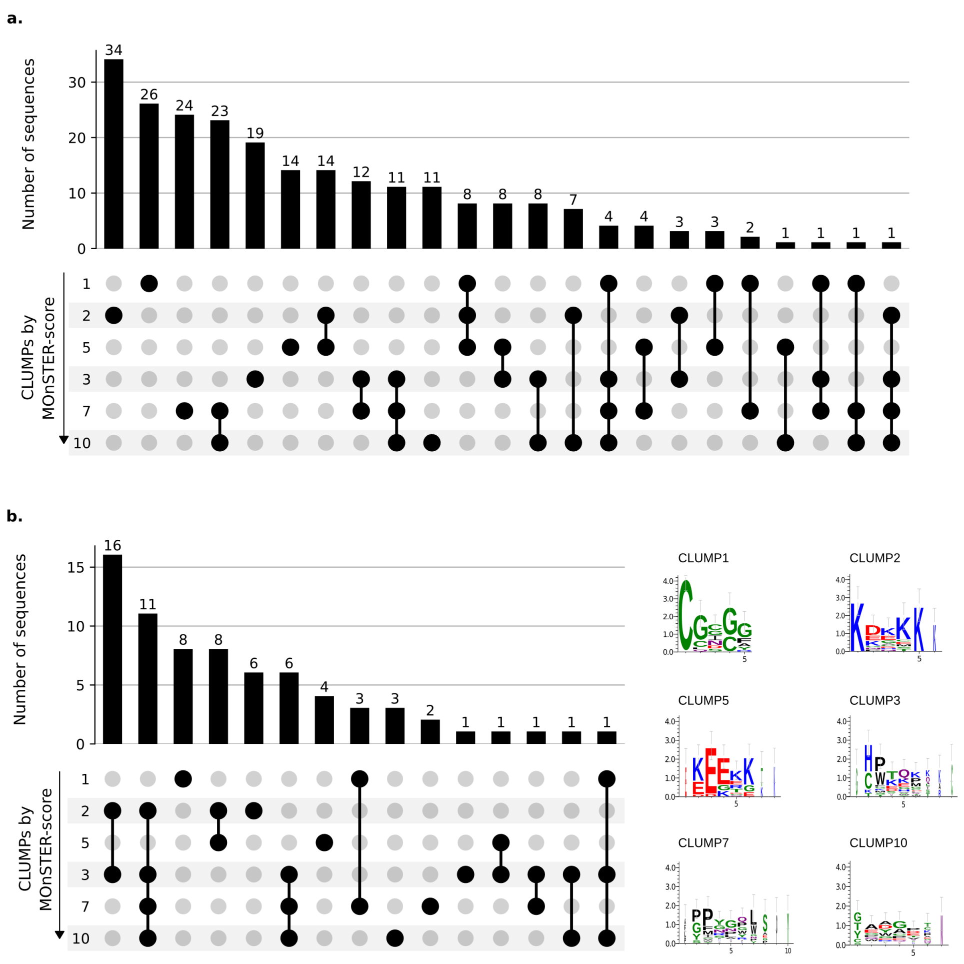

Representation of occurrence and co-occurrence of each best-scoring CLUMP in protein sequences, according to their MOnSTER-score. **(a)** shows the number of sequences and the occurrence of CLUMPs in the sequences bearing the signal peptide, while **(b)** represents the same measures in the sequences without the signal peptide. In the bottom right corner, the motif logos of the respective CLUMPs (colors corresponds to 'chemistry' color-scheme in WebLogo3 [1] used to obtain the images).

Supplementary Figure 6: Relation between CLUMPs abundance and protein sequence length – PPNs.

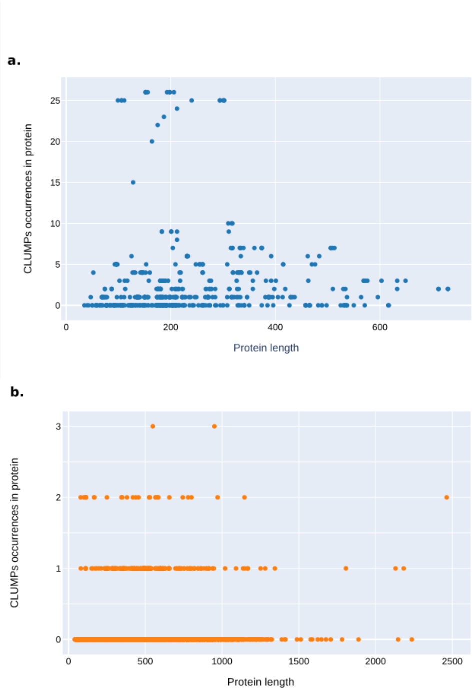

(**a**) Shows a non-linear relationship between the occurrences of CLUMPs and the length of sequences in the positive dataset, while **(b)** represents the same but in the negative dataset.

Supplementary Figure 7: Protein sequence position of motifs in CLUMP7 and 10 associated with glycosyl hydrolase family 5 domain (dc2).

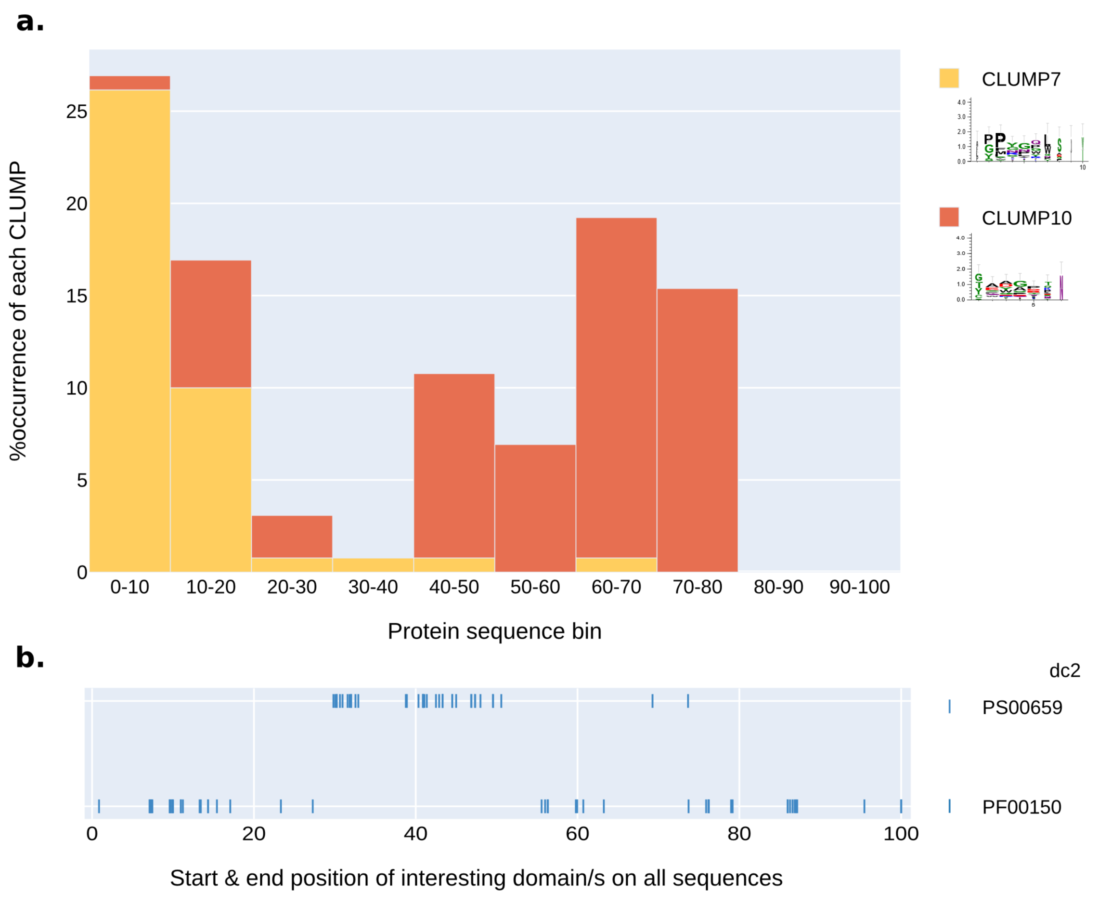

(a) CLUMP7 and CLUMP10 motifs-position and respective occurrence along selected protein sequences divided into bins representing 10% sequence portion. **(b)** rug-plot representing the start and end position of the domains (PS00659-ProSitePatterns and PF00150-Pfam) belonging to the class 2 of domains, namely glycosyl hydrolase family 5 domains. In the upper right corner, the motif logos of the respective CLUMPs (colors corresponds to 'chemistry' color-scheme in WebLogo3 [1] used to obtain the images).

Supplementary Figure 8: PRO-MOCA algorithm.

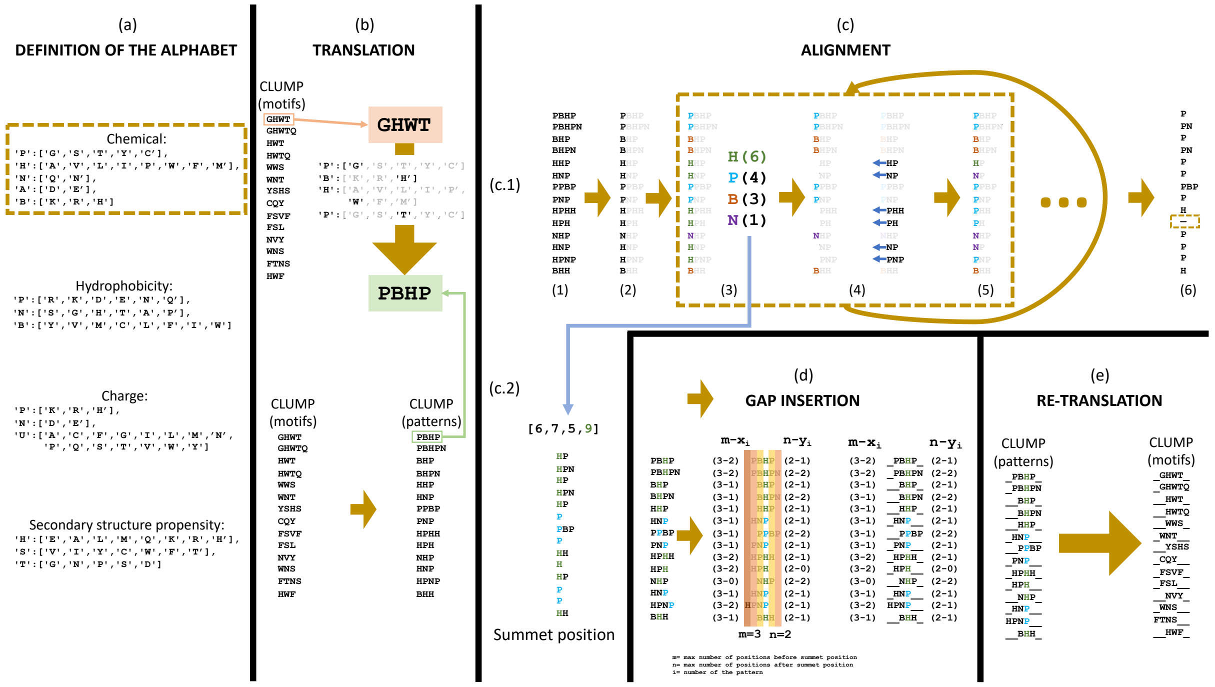

(a) DEFINITION OF THE ALPHABET. The user defines the alphabet to use to translate the protein sequences into patterns. Alphabet are constructed as pairs of keys, a letter defined by the user, and values, a list of amino acids to include in the group. Amino acids from the group will be replaced by the key in the sequence. PRO-MOCA already include the following alphabets. Chemical alphabet: ‘P’ = ’polar’, ‘H’ = hydrophobic, ‘N’= ‘neutral’, ‘A’ = ’acid’, ‘B’ = ‘basic’. Hydrophobicity alphabet: ‘P’ = ‘hydrophilic’, ’N’ = ‘neutral’, ‘B’ = ‘hydrophobic’. Charge alphabet: ‘P’ = ’positive’, ‘N’ = ’negative’, ’U’ = ‘neutral’. Secondary structure propensity alphabet: ‘H’ = ‘helix (α)’, ‘S’ = ‘sheet (β)’, ‘T’ = ‘turn’. **(b) TRANSLATION.** Using the chosen alphabet (step (a)), the motifs in the CLUMP are translated from the protein alphabet (amino acids) into patterns of the given alphabet. For instance, using the ‘chemical’ alphabet, the ‘GHWT’ motif is translated into ‘PBHP’. **(c) ALIGNMENT.** (c1) PRO-MOCA: (1) takes the list of patterns, (2) selects the first position of each pattern, (3) calculates how many times each letter is repeated at that position and finds the most repeated letter (PRO-MOCA stores this result at each iteration) (4) After that, patterns with the most repeated letter at the first position, lose that letter, and the rest of the pattern is shifted of -1 position. (5) The result is a list of patterns where part of the patterns has a new first position. From this point, the process (3)-(5) is repeated until (6) the result is a list where at least one pattern does not have any more letters. Until there is a pattern with no more letters (golden rectangle). (c2) Once the first part of the alignment is done and PRO-MOCA gets to (6), it finds the central position of the alignment. To do that, PRO-MOCA stored in (3) the number of repetitions of the most repeated letter. In (c2) PRO-MOCA calculates which is the highest value, and gets the corresponding list of patterns with those first positions. **(d) GAP INSERTION**. The gap insertion is only allowed at the extremities of the patterns and allows to have an alignment where all the patterns have the same length. To do that, PRO-MOCA calculates, for each pattern, how many letters there are before (xi) and after (yi) the central position and calculates the maximum (m and n) of these values. After that, it subtracts m – xi and n – yi. The result of the subtraction is the number of gaps each pattern has

before and after the central position. **(e) RE-TRANSLATION.** In this step, PRO-MOCA re-translates the aligned patterns into aligned motifs.

Supplementary Tables

Supplementary Table 1: Oomycetes' MonSTER-score.

CLUMP	monster_score	# motifs	occurrences_pos_dset %	occurrences_neg_dset %
7	1,987480543	22	0,180149168	0,002991027
4	1,899071502	31	0,162937464	0,003988036
10	1,632604583	10	0,393000574	0,117647059
6	1,62987555	32	0,227768216	0,030907278
2	1,612504831	26	0,229489386	0,011964108
9	1,525031887	13	0,045897877	0,001329345
5	1,286201078	28	0,128514056	0,000332336
0	1,230544758	37	0,185886403	0,005649718
1	1,078631508	24	0,1589214	0,009305417
3	0,766727747	29	0,117039587	NaN
8	0	13	0,104991394	NaN

Summary table of MOnSTER-score for each CLUMP found in oomycetes dataset, ranked by higher score.

Columns: CLUMP_ID, assigned MOnSTER-score, number of motifs in the CLUMP, occurrences of CLUMP-Motifs in the positive and the negative dataset.

Supplementary Table 2: PPNs' MOnSTER-score.

CLUMP	monster_score	# motifs	occurrences_pos_dset %	occurrences_neg_dset %
1	1,992942246	17	0,104395604	0,000779423
2	1,728641386	15	0,197802198	0,024941543
5	1,721815487	20	0,119047619	0,024941543
3	1,621936233	24	0,192307692	0,00233827
7	1,543503351	28	0,194139194	0,003377501
10	1,430906419	30	0,163003663	0,003117693
8	1,175646331	23	0,186813187	0,002598077
9	0,989616653	32	0,245421245	0,003637308
6	0,249649712	25	0,188644689	NaN
4	0,202119994	28	0,188644689	NaN
0	0,156226039	27	0,120879121	NaN

Summary table of MOnSTER-score for each CLUMP found in PPNs dataset, ranked by higher score.

Columns: CLUMP_ID, assigned MOnSTER-score, number of motifs in the CLUMP, occurrences of CLUMP-Motifs in the positive and the negative dataset.

Supplementary Table 3: *Meloidogyne incognita* proteins for validation of putative effector.

Seq_id	1	2	3	5	7	10	has_SP	nb_TM	SPnoTM	Cluster	Mel-spec	Max-2copies	CLUMP motif	Sequence length	Start pos on seq	Interpro proximity domain
Minc3s00008g00521	0	0	0	2	0	0	1	0	1	D	Y	Y				
Minc3s00023g01445	0	0	0	1	0	0	1	0	1	A	Y	Y				
Minc3s00056g02931	0	0	0	1	0	0	1	0	1	F	Y	Y	GEEKK	352	53	Disorder prediction mobidb-lite
Minc3s00420g12033	0	0	0	2	0	0	1	0	1	D	Y	Y				
Minc3s00618g15179	0	0	0	1	0	0	1	0	1	D	Y	Y				
Minc3s01189g21535	0	0	0	2	0	0	1	0	1	D	Y	Y				
Minc3s01206g21700	0	0	0	1	0	0	1	0	1	G	Y	Y				
Minc3s02686g31190	0	0	0	2	0	0	1	0	1	D	Y	Y				
Minc3s00034g02036	0	0	0	1	0	0	1	0	1	C	Y	-				
Minc3s00160g06403	0	0	0	1	0	0	1	0	1	A	Y	-				
Minc3s00275g09183	0	0	0	1	0	0	1	0	1	A	Y	-				
Minc3s00610g15098	0	0	0	2	0	0	1	0	1	C	Y	-				
Minc3s02752g31449	0	0	0	1	0	0	1	0	1	H	Y	-				
Minc3s03183g33074	0	0	0	1	0	0	1	0	1	A	Y	-				
Minc3s00088g04182	0	0	0	1	0	0	1	0	1	H	NN	-				
Minc3s00164g06507	0	0	0	2	0	0	1	0	1	G	NN	-				
Minc3s00091g04260	0	0	0	1	0	0	1	0	1	D	NN	-				
Minc3s00098g04484	0	0	0	1	0	0	1	0	1	A	NN	-				
Minc3s00175g06778	0	0	0	1	0	0	1	0	1	G	NN	-				
Minc3s00193g07189	0	0	0	1	0	0	1	0	1	F	NN	-				
Minc3s00343g10611	0	0	0	2	0	0	1	0	1	A	NN	-				
Minc3s00676g15957	0	0	0	1	0	0	1	0	1	B	NN	-				
Minc3s00861g18200	0	0	0	1	0	0	1	0	1	D	NN	-				
Minc3s01000g19730	0	0	0	1	0	0	1	0	1	B	NN	-				
Minc3s01196g21604	0	0	0	1	0	0	1	0	1	A	NN	-				
Minc3s01269g22302	0	0	0	2	0	0	1	0	1	E	NN	-				
Minc3s01327g22812	0	0	0	2	0	0	1	0	1	E	NN	-				
Minc3s01338g22906	0	0	0	3	0	0	1	0	1	H	NN	-				
Minc3s01380g23311	0	0	0	1	0	0	1	0	1	G	NN	-				
Minc3s01460g24007	0	0	0	2	0	0	1	0	1	E	NN	-				
Minc3s01492g24241	0	0	0	1	0	0	1	0	1	E	NN	-				
Minc3s01614g25114	0	0	0	1	0	0	1	0	1	D	NN	-				
Minc3s01699g25717	0	0	0	1	0	0	1	0	1	B	NN	-				
Minc3s01725g25884	0	0	0	1	0	0	1	0	1	D	NN	-				

Seq_id	1	2	3	5	7	10	has_SP	nb_TM	SPnoTM	Cluster	Mel-spec	Max-2copies	CLUMP motif	Sequence length	Start pos on seq	Interpro proximity domain
Minc3s01785g26287	0	0	0	1	0	0	1	0	1	B	NN	-				
Minc3s02254g29093	0	0	0	1	0	0	1	0	1	D	NN	-				
Minc3s02607g30865	0	0	0	1	0	0	1	0	1	H	NN	-				
Minc3s02665g31118	0	0	0	2	0	0	1	0	1	H	NN	-				
Minc3s02667g31120	0	0	0	1	0	0	1	0	1	F	NN	-				
Minc3s03121g32864	0	0	0	1	0	0	1	0	1	F	NN	-				
Minc3s03360g33617	0	0	0	2	0	0	1	0	1	D	NN	-				
Minc3s03408g33756	0	0	0	2	0	0	1	0	1	D	NN	-				
Minc3s03844g34961	0	0	0	1	0	0	1	0	1	H	NN	-				
Minc3s04356g36102	0	0	0	1	0	0	1	0	1	H	NN	-				
Minc3s04535g36491	0	0	0	1	0	0	1	0	1	A	NN	-				
Minc3s05314g38002	0	0	0	1	0	0	1	0	1	E	NN	-				
Minc3s05318g38010	0	0	0	1	0	0	1	0	1	D	NN	-				
Minc3s05347g38060	0	0	0	1	0	0	1	0	1	F	NN	-				
Minc3s05357g38086	0	0	0	1	0	0	1	0	1	C	NN	-				
Minc3s09122g42949	0	0	0	1	0	0	1	0	1	H	NN	-				
Minc3s00089g04218	0	0	0	3	0	0	1	0	1	C	N	-				
Minc3s00126g05384	0	0	0	1	0	0	1	0	1	C	N	-				
Minc3s00552g14177	0	0	0	1	0	0	1	0	1	D	N	-				
Minc3s00895g18614	0	0	0	1	0	0	1	0	1	D	N	-				
Minc3s01331g22845	0	0	0	2	0	0	1	0	1	C	N	-				
Minc3s02925g32084	0	0	0	1	0	0	1	0	1	C	N	-				

List of proteins in *M. incognita* proteome presenting at least one motif belonging to CLUMP5 (the most abundant in the proteome) and predicted to possess signal peptide and no transmembrane domain. Further details on the selection criteria can be found in the “Results & Discussion” section of the main text.

Columns: sequence ID, number of motifs per significative CLUMP (1 column each), presence of Signal Peptide and absence of Transmembrane Domains (3 columns), expression cluster by Da Rocha et al., 2021 [2], M. incognita candidates that had no homologs in other species than the RKNs (NN=homologs are present in other nematodes also not plant-parasitic species; N=homologs are present in other species of PPN other than M. incognita genus, Y=true), M.incognita genes that were in maximum 2 copies (Y=true, ‘-’ = more than 2 copies), CLUMP5 motif of the validated putative effector, length of the protein, start position of motif in the protein, InterProScan identified domain.

Supplementary Data 1: Single file containing all the data related to main figures/graphs.

Each sheet in the file is named accordingly to the order of the main figures. Sheet 1.5: InterProScan domains and CLUMPs co-occurrences.

Supplementary Data 2.1_2.2: Oomycetes' positive and negative datasets.

Detailed description of the protein sequences in the oomycetes' positive and negative datasets including species, retrieving sources, and corresponding amino acid sequence.

Supplementary Data 3.1_3.2: PPNs' positive and negative datasets.

Detailed description of the protein sequences in the PPNs' positive and negative datasets including species, retrieving sources, and corresponding amino acid sequence.

Supplementary References

1. Crooks, G. E., Hon, G., Chandonia, J.-M. & Brenner, S. E. WebLogo: a sequence logo generator. *Genome Res* **14**, 1188–1190 (2004).
2. Da Rocha, M. *et al.* Genome Expression Dynamics Reveal the Parasitism Regulatory Landscape of the Root-Knot Nematode Meloidogyne incognita and a Promoter Motif Associated with Effector Genes. *Genes (Basel)* **12**, 771 (2021).
