# Supplementary material for: Identification and characterization of specific motifs in effector proteins of plant parasites using MOnSTER
Source: Commun Biol. 2024 Jul 12;7:850. doi: 10.1038/s42003-024-06515-9 (PMC11239862; doi:10.1038/s42003-024-06515-9)
Supplement: Supplementary file 3 — Description of Additional Supplementary Files [file 42003_2024_6515_MOESM3_ESM.pdf]

Description of Additional Supplementary Files

File name: Supplementary Data1.1
Description: PROMOCA alignments for selected CLUMPs (10, 2, 4, 7) from MOnSTER oomycetes application

File name: Supplementary Data1.2
Description: PROMOCA alignments for best scoring CLUMPs (10, 2, 4, 7, 6, 9) from MOnSTER oomycetes application

File name: Supplementary Data1.3
Description: PROMOCA alignments for best scoring CLUMPs (1, 2, 5, 3, 7, 10) from MOnSTER plant parasitic nematodes application

File name: Supplementary Data1.4
Description: CLUMPs per plant parasitic nematodes species.

File name: Supplementary Data1.5
Description: InterProScan domains and CLUMPs co-occurrences.

File name: Supplementary_Data1.6
Description: Characteristics of recently published effectors of M. incognita, and the new one.

File name: Supplementary_Data2.1
Description: Detailed description of the protein sequences in the oomycetes' positive dataset including species, retrieving sources, and corresponding amino acid sequence.

File name: Supplementary_Data2.2
Description: Detailed description of the protein sequences in the oomycetes' negative dataset including species, retrieving sources, and corresponding amino acid sequence.

File name: Supplementary_Data3.1
Description: Detailed description of the protein sequences in the PPNs' positive dataset including species, retrieving sources, and corresponding amino acid sequence.

File name: Supplementary_Data3.2
Description: Detailed description of the protein sequences in the PPNs' negative dataset including species, retrieving sources, and corresponding amino acid sequence.
