# Supplementary material for: Identification and characterization of specific motifs in effector proteins of plant parasites using MOnSTER
Source: Commun Biol. 2024 Jul 12;7:850. doi: 10.1038/s42003-024-06515-9 (PMC11239862; doi:10.1038/s42003-024-06515-9)
Supplement: Supplementary file 7 — reporting summary [file 42003_2024_6515_MOESM7_ESM.pdf]

nature portfolio

Corresponding author(s): Silvia Bottini

Last updated by author(s): 29/05/24

Reporting Summary

Nature Portfolio wishes to improve the reproducibility of the work that we publish. This form provides structure for consistency and transparency in reporting. For further information on Nature Portfolio policies, see our Editorial Policies and the Editorial Policy Checklist.

Statistics

For all statistical analyses, confirm that the following items are present in the figure legend, table legend, main text, or Methods section.

n/a	Confirmed	
☐	☒	The exact sample size (*n*) for each experimental group/condition, given as a discrete number and unit of measurement
☒	☐	A statement on whether measurements were taken from distinct samples or whether the same sample was measured repeatedly
☐	☒	The statistical test(s) used AND whether they are one- or two-sided<br>*Only common tests should be described solely by name; describe more complex techniques in the Methods section.*
☒	☐	A description of all covariates tested
☐	☒	A description of any assumptions or corrections, such as tests of normality and adjustment for multiple comparisons
☐	☒	A full description of the statistical parameters including central tendency (e.g. means) or other basic estimates (e.g. regression coefficient) AND variation (e.g. standard deviation) or associated estimates of uncertainty (e.g. confidence intervals)
☐	☒	For null hypothesis testing, the test statistic (e.g. *F*, *t*, *r*) with confidence intervals, effect sizes, degrees of freedom and *P* value noted<br>*Give P values as exact values whenever suitable.*
☒	☐	For Bayesian analysis, information on the choice of priors and Markov chain Monte Carlo settings
☒	☐	For hierarchical and complex designs, identification of the appropriate level for tests and full reporting of outcomes
☒	☐	Estimates of effect sizes (e.g. Cohen's *d*, Pearson's *r*), indicating how they were calculated

Our web collection on statistics for biologists contains articles on many of the points above.

Software and code

Policy information about availability of computer code

Data collection	Oomycetes dataset:<br>1743 proteins (positive dataset) from both PHI-base database (v4.14), Uniprot (release 2023_02) and publication Haas et al., (2009) [doi:10.1038/nature08358].<br>3009 proteins negative dataset) from Uniprot (2023_02).<br>Uniprot entries derived from five oomycete species, namely, Phytophthora infestans,<br>Phytophthora sojae, Phytophthora ramorum, Hyaloperonospora arabidopsidis and<br>Bremia lactucae.<br><br>Plant Parasitic Nematodes dataset:<br>546 proteins (positive dataset) from NCBI GeneBank via NCBI 'entrez' API, WormBase ParaSite (vWBPS17-WS282) and nematode.net (v4.0) websites.<br>3849 proteins (negative dataset) filtering of results from previous analysis in P. Grynberg et al., (2020) [doi: 10.3390/genes11111347].<br>A total of 13 PPN species are considered Meloidogyne incognita, Meloidogyne javanica, Meloidogyne arenaria, Meloidogyne hapla, Meloidogyne chitwoodi, Meloidogyne graminicola, Globodera rostochiensis, Globodera pallida, Heterodera havenae, Heterodera glycines, Heterodera schachtii, Radopholus similis, Bursaphelenchus xylophilus.
Data analysis	Oomycetes dataset:<br>Protein filtering: in both positive and negative dataset with CD-HIT (v4.8.1).<br>Negative dataset selection refinition: Orthofinder (v2.5.4).<br>Motif discovery: STREME (v5.5.1), FIMO (v5.5.1), MERCI (v1).

Plant Parasitic Nematodes dataset:
Protein filtering: CD-HIT-2D and CD-HIT (v4.8.1) intra- and inter- species for both positive and negative datasets.
Motif discovery: STREME (v5.5.1), FIMO (v5.5.1), MERCI (v1).
MOnSTER code is publicly available on GitHub at https://github.com/paolaporracciolo/MOnSTER_PROMOCA.git under the GNU GENERAL PUBLIC LICENSE (v3.0, 29-06-2007). All dependencies can be installed directly by command line 'pip install -e /path/to/MOnSTER' as describe in the README file for the repository.
Motif logo of CLUMPs by MOnSTER: Weblogo (v3).
Protein domains and signal peptide mining analysis: InterProScan (V5.54-87.0), SignalP (v4.1), TMHMM (v2.0).
In situ hybridization of M. incognita coding sequence protocol follows the previously described ones in M.-C. Caillaud and B. Favery, 2016 [doi: 10.1007/978-1-4939-3142-2_11], and in M. Jaouannet et al., (2018) [doi: 10.21769/BioProtoc.2766].
Custom scripts for data analysis throughout the study used Python programming language (v3.8) and the following libraries:
pandas (v1.2.4)
numpy (1.21.2)
UpSetPlot (v0.8.0)
matplotlib (v3.7.1)
scipy (v1.10.1)
radialtree (v0.1.0)
re (v2.2.1)
Bio (Biopython) (v1.70)
plotly (v5.10.0)
sklearn (v1.1.1)

For manuscripts utilizing custom algorithms or software that are central to the research but not yet described in published literature, software must be made available to editors and reviewers. We strongly encourage code deposition in a community repository (e.g. GitHub). See the Nature Portfolio guidelines for submitting code & software for further information.

Data

Policy information about availability of data

All manuscripts must include a data availability statement. This statement should provide the following information, where applicable:

- Accession codes, unique identifiers, or web links for publicly available datasets
- A description of any restrictions on data availability
- For clinical datasets or third party data, please ensure that the statement adheres to our policy

The source code and related data are available at: https://github.com/Plant-Net/MOnSTER_PROMOCA.git

Research involving human participants, their data, or biological material

Policy information about studies with human participants or human data. See also policy information about sex, gender (identity/presentation), and sexual orientation and race, ethnicity and racism.

Reporting on sex and gender	This research does not involve human participants, their data or biological material
Reporting on race, ethnicity, or other socially relevant groupings	This research does not involve human participants, their data or biological material
Population characteristics	NA
Recruitment	NA
Ethics oversight	NA

Note that full information on the approval of the study protocol must also be provided in the manuscript.

Field-specific reporting

Please select the one below that is the best fit for your research. If you are not sure, read the appropriate sections before making your selection.

☒ Life sciences ☐ Behavioural & social sciences ☐ Ecological, evolutionary & environmental sciences

For a reference copy of the document with all sections, see nature.com/documents/nr-reporting-summary-flat.pdf

Life sciences study design

All studies must disclose on these points even when the disclosure is negative.

Sample size	For each ISH experiment (each probe), 10,000 Meloidogyne incognita juvenile (J2) were used as described in Jaouannet et al. 2018 (Bioprotocol). This number means that at least a hundred nematodes can be observed at the end of the experiment, despite losses during the

various treatments. For Nicotiana benthamiana agroinfiltration, a minimum of three leaves were agroinfiltrated and observed for the construct (or water control) in each experiment in order to take into account the possible variability of expression between leaves.

Data exclusions	No data were excluded from the analysis.
Replication	ISH with the antisense probe were carried out three times independently and 30 pictures were taken; ISH with the sense negative probe were carried once and 12 pictures were taken; Agroinfiltrations with the GFP fusion or negative control (water) were carried out three times independently and 25 and 13 pictures were taken, respectively. All attempts at replication were successful, i.e. signals observed for ISH antisense probes or GFP fusion for agroinfiltration, or not in the case of negative controls (sense probe or water infiltration, respectively).
Randomization	There was no experimental groups in this study as no comparison between conditions were done.
Blinding	Blinding was not relevant for this study because no factor related to sex or age or other factor could influence study results.

Reporting for specific materials, systems and methods

We require information from authors about some types of materials, experimental systems and methods used in many studies. Here, indicate whether each material, system or method listed is relevant to your study. If you are not sure if a list item applies to your research, read the appropriate section before selecting a response.

Materials & experimental systems

n/a	Involved in the study
☒	☐ Antibodies
☒	☐ Eukaryotic cell lines
☒	☐ Palaeontology and archaeology
☐	☒ Animals and other organisms
☒	☐ Clinical data
☒	☐ Dual use research of concern
☐	☒ Plants

Methods

n/a	Involved in the study
☒	☐ ChIP-seq
☒	☐ Flow cytometry
☒	☐ MRI-based neuroimaging

Animals and other research organisms

Policy information about studies involving animals; ARRIVE guidelines recommended for reporting animal research, and Sex and Gender in Research

Laboratory animals	We used root-knot nematodes (Meloidogyne incognita strain Morelos from our INRAE collection of root-knot nematodes multiplied in Solanum lycopersicum St Pierre).
Wild animals	The study did not involve wild animals.
Reporting on sex	These species are parthenogenetic with only females participating to the offspring.
Field-collected samples	The study did not involve samples collected from the field.
Ethics oversight	No ethical approval or guidance was required for this study as it concerns genome analysis of organisms reared in an INRAE nematode collection.

Note that full information on the approval of the study protocol must also be provided in the manuscript.

Dual use research of concern

Policy information about dual use research of concern

Hazards

Could the accidental, deliberate or reckless misuse of agents or technologies generated in the work, or the application of information presented in the manuscript, pose a threat to:

No	Yes	
☒	☐	Public health
☒	☐	National security
☒	☐	Crops and/or livestock
☒	☐	Ecosystems
☒	☐	Any other significant area

Experiments of concern

Does the work involve any of these experiments of concern:

No	Yes	
☒	☐	Demonstrate how to render a vaccine ineffective
☒	☐	Confer resistance to therapeutically useful antibiotics or antiviral agents
☒	☐	Enhance the virulence of a pathogen or render a nonpathogen virulent
☒	☐	Increase transmissibility of a pathogen
☒	☐	Alter the host range of a pathogen
☒	☐	Enable evasion of diagnostic/detection modalities
☒	☐	Enable the weaponization of a biological agent or toxin
☒	☐	Any other potentially harmful combination of experiments and agents

Plants

Seed stocks	Seed stock of Nicotiana benthamiana (wild-type) for agroinfiltration; seed stock of Solanum lycopersicum ('St Pierre' cultivar) for nematode multiplication.
Novel plant genotypes	NA
Authentication	NA
